# A decade of invertebrate recruitment at Santa Catalina Island, California

**DOI:** 10.7717/peerj.14286

**Published:** 2022-11-08

**Authors:** Peter J. Edmunds, Jessica Clayton

**Affiliations:** Department of Biology, California State University, Northridge, Northridge, CA, United States

**Keywords:** Monitoring, Recruitment, Long-term, Change, Subtidal, Marine

## Abstract

Marine fouling communities have long provided model systems for studying the ecology of community development, and settlement plates are the tool of choice for this purpose. Decades of plate deployments provide a baseline against which present-day trends can be interpreted, with one classic trend being the ultimate dominance of plates by colonial and encrusting taxa. Here we report the results of annual deployments of settlement plates from 2010 to 2021 in the shallow sub-tidal of southern California, where the recruitment of invertebrates and algae was recorded photographically, and resolved to functional group (solitary, encrusting, and arborescent) and the lowest taxon possible. The communities on these plates differed among years, with trends in abundances varying by functional group and taxon; solitary taxa consistently were abundant, but encrusting taxa declined in abundance. Seawater temperature and the subsurface concentration of chlorophyll a differed among years, and there was a weak inverse association between temperature and the abundances of encrusting taxa. Long-term increases in seawater temperature therefore could serve as a mechanism causing fouling communities to change. Because of the prominence of encrusting taxa in fouling communities, the shifts in abundance of this functional group reported here may portend ecologically significant changes in fouling communities exposed to warmer seawater because of an alleviation of competition for a classically limiting resource (*i.e*., space).

## Introduction

Benthic communities on hard surfaces in the marine environment (*i.e*., fouling communities (Richmond & Seed, 1991)) have been quantitatively studied for more than a century (McDougall, 1943), and the results have played important roles in understanding the ecological processes determining community development (Sutherland, 1974; Sutherland & Karlson, 1977; Buss & Jackson, 1979). Settlement plates are the favored tool in such analyses, and they have been made of an assortment of materials in a variety of sizes, and typically are immersed for a range of times, at different orientations, and a variety of depths. While settlement plates are one of the oldest and most basic of tools in marine ecology (*e.g*., McDougall, 1943), they are enjoying a resurgence of use to understand a rapidly changing marine environment (Kordas et al., 2015; Obst et al., 2020).

Settlement plates do not accurately mimic natural benthic surfaces because they usually are fabricated from ecologically irrelevant materials (*e.g*., PVC (Zimmerman & Martin, 2004; Romain et al., 2019)), and lack the rugosity and biological complexity of natural surfaces (but see Thomason et al., 2002; Nozawa, Tanaka & Reimer, 2011). Nonetheless, settlement plates provide standardized tools that serve as assays for recruitment and community development to support relative contrasts among sites and times (Thomason et al., 2002; Obst et al., 2020). Most deployments of settlement plates last only a few years, and often are tied to the length of graduate degrees. While substantial temporal variation in the communities developing on settlement plates usually is revealed by even short deployments (*e.g*., Bram, Page & Dugan, 2005), such deployments limit the capacity to interpret variation, or to ascribe causation. Over the last 70 years, settlement plates deployed in a statistically robust framework have advanced ecological theory in multiple areas (*e.g*., Sutherland, 1974; Jackson, 1977; Sutherland & Karlson, 1977; Buss & Jackson, 1979) and, most recently, in testing for variation associated with anthropogenic disturbances and climate change (Kordas et al., 2015; Obst et al., 2020).

Once immersed, settlement plates are colonized by a variety of animals and algae, but the diversity of taxa involved varies among locations, for example, being greater in the tropics *vs*. temperate locations (Bracewell, Clark & Johnston, 2018). Regardless of the regional species pool from which the recruits arise, a successional sequence can emerge, with solitary taxa among the earliest recruits, and colonial and encrusting organisms dominating later (Jackson, 1977). As space becomes occupied, competition for this limited resource intensifies, and competitive hierarchies and transitive networks emerge (Buss & Jackson, 1979). The generalities of these interactions led JBC Jackson to contrast the adaptive benefits of solitary and colonial strategies in spatial competition on benthic surfaces (Jackson, 1977). He noted that solitary organisms (*e.g*., polychaetes, and cirripedes) generally do not proliferate by asexual budding, and exhibit “simple” growth (*i.e*., increase in body size) with enclosing exoskeletons, whereas colonial organisms (*e.g*., sponges and bryozoans) expand asexually through modular proliferation, and usually lack restrictive exoskeletons. Jackson (1977), therefore, highlighted the potential of colonial taxa as superior space competitors capable of dominating surfaces, unless growth is interrupted by disturbances (terHorst & Dudgeon, 2009). Against this backdrop, present-day environmental conditions that differentially affect solitary *vs*. colonial taxa are likely to modulate the structure of marine communities developing on hard surfaces.

Decades of work on recruitment in marine communities, especially into fouling communities, have revealed the complexity of events determining where, when, and which taxa dominate hard surfaces (*e.g*., Sutherland & Karlson, 1977; Richmond & Seed, 1991). There also have been large efforts to understand the biophysical drivers of variation in recruitment and post-settlement success in these systems (see Menge et al., 2011 for one elegant example of this approach), and from this work it has emerged that seawater temperature and food supply are particularly important (Pineda, Reyns & Starczak, 2009; Menge et al., 2011). Temperature has pervasive and profound effects on organism performance (Brown et al., 2004; Angilletta, 2009), and with respect to recruitment to hard surfaces in the marine environment, modulates the delivery of pelagic propagules (O’Connor et al., 2007) and the development of marine communities (Bruno, Carr & O’Connor, 2015). The well-known effects of temperature on organism performance (Somero, 2002, 2012) indicate that that the species of organisms recruiting to hard surfaces will respond in dissimilar ways to temperature. Variation in seawater temperature, therefore, can be expected to drive changes in the communities that develop on hard surfaces (*e.g*., Sorte, Williams & Zerebecki, 2010), with these effects expected to be profound if members of functionally important groups are effected in similar ways. Such outcomes could arise, for example, if changes in temperature differentially affect early successional stages, or the taxa that historically have been dominant spatial competitors (like colonial/encrusting taxa; Jackson, 1977).

This study describes changes in the fouling community on hard surfaces in the shallow sub-tidal of southern California. Recruitment was assayed using settlement tiles immersed beneath a dock at Santa Catalina Island for 10 samplings each lasting approximately 1 year. On retrieval, the lower surfaces of the tiles were photographed to quantify the fouling community, with an emphasis on invertebrates. Algae were also recorded, and while they contributed to the arborescent functional group, they are not addressed in detail. The abundances of invertebrates separated to solitary and colonial/encrusting functional groups were used to: (1) test the hypothesis that abundances of these groups, and their members, have changed, and (2) test for associations between invertebrate recruitment and select environmental conditions to gain insight into causation of the changes.

## Methods

### Biological sampling

Unglazed terracotta tiles (19.3 cm × 19.3 cm × 1 cm) were used to measure recruitment at yearly intervals over 10 years from 19 November 2010 to 30 March 2021 (Fig. S1, Supplemental Material). Tiles were deployed rough side downward, with each deployment consisting of 14–19 tiles mounted on a single stainless steel rod (91 cm long) that was passed through a hole drilled in the center of each tiles. Tiles were loaded on the rod with plastic spacers keeping tiles ~1 cm apart and creating a cryptic habitat favored by recruits. The stainless steel rod and mounted tiles was suspended beneath the stationary dock (*i.e.*, non-floating) of the Wrigley Institute for Environmental Studies (WIES). Tiles were located at ~2 m depth (near low tide), but depth varied with tidal state (greatest range in this locations is ~2.7 m). For 3 years (immersion years from January 2016 to February 2019), the assembly was deployed at a fixed depth (~2 m) beneath a floating dock attached to the end the stationary dock, with the tiles ~15 m northwest of their initial position. Preliminary analysis of the mean abundances of taxa by year for the two deployment configurations (*n* = 3 years on the floating dock *vs. n* = 7 years on the static dock) showed no difference in abundance for solitary, encrusting, and arborescent taxa (*P* ≥ 0.149, Supplemental Material).

Tiles were retrieved and replaced on the same day ~1 year after deployment. Freshly collected tiles were removed from the stainless rod and photographed with a Nikon DSLR that was upgraded throughout the project to provide images varying in size from 12 to 36 MP. The top and bottom of the tiles were recorded in natural sunlight, and the tiles preserved in 90% ethanol to provide reference materials to enhance enumeration and support outreach activities in schools. The next batch of tiles was loaded onto the same stainless steel rod that was immersed until the following retrieval. Prior to the subsequent sampling, the preserved tiles were scraped clean, soaked for several days in fresh water, and returned to Santa Catalina Island for the next tile exchange.

Following approximately a year of immersion, the tiles were encrusted with animals and algae, with little free space remaining. Photography was used to record the organisms, but did not provide sufficient resolution to identify most organisms to species, or to count organisms in layered communities. Preliminary screening of the tiles was used to develop a consensus list of organisms that could be identified to taxon. Analyses focused on the lower surface of the tiles, as upper surfaces were prone to sediment accumulation that smothered settlers and reduced photographic resolution. Organisms were resolved to solitary, colonial/encrusting, and arborescent functional groups. Initially, analyses identified 35 solitary and seven colonial/encrusting taxa, but arborescent groups proved unresolvable. Based on the inability to identify the full size range of organisms on the tiles (small organisms were challenging), taxa were upwardly pooled to the lowest taxonomic rank that could reliably be resolved. Sixteen solitary, six encrusting groups, and a functional group combined as “arborescent” were resolved, with the arborescent taxa consisting of bryozoans, hydroids, and articulated coralline algae (Table 1).

**Table 1:** Summary of consensus resolution used to score organisms on settlement tiles. The capacity to resolve large specimens of many groups was greater than indicated in this table, but in such cases taxonomy became uncertain with smaller specimens necessitating a coarser consensus taxonomic resolution.

Functional group	Taxon scored	Phylum	Class	Sub class	Order	Family
Solitary	*Chaetopterus variopedatus*	Annelida	Polychaeta	Sedentaria	n/a	Chaetopteridae
Solitary	*Phragmatopoma californica*	Annelida	Polychaeta	Sedentaria	n/a	Sabellariidae
Solitary	Polynoidae	Annelida	Polychaeta	Errantia	Phyllodocida	Polyneoidae
Solitary	*Spirobranchus* spp.	Annelida	Polychaeta	Sedentaria	Sabellida	Serpulidae
Solitary	*Spirorbis* spp.	Annelida	Polychaeta	Sedentaria	Sabellida	Serpulidae
Solitary	Vermetidae	Mollusca	Gastropoda	Coenogastropoda	Littorinimorpha	Vermetidae
Solitary	Cirrepedia	Arthropoda	Thecostraca	Cirripedia	n/a	n/a
Solitary	Eumalacostraca	Arthropoda	Malacostraca	Eumalacostraca	n/a	n/a
Solitary	Ascidiacea	Chordata	Ascidiacea	n/a	n/a	n/a
Solitary	Ophiuroidea	Echinodermata	Ophiuroidea	n/a	n/a	n/a
Solitary	Gastropoda	Mollusca	Gastropoda	n/a	n/a	n/a
Solitary	Bivalvia	Mollusca	Bivalvia	n/a	n/a	n/a
Solitary	*Aplysia* spp.	Mollusca	Gastropoda	Heterobranchia	Aplysiida	Asplysiidae
Solitary	*Tegula* spp.	Mollusca	Gastropoda	Vetigastropoda	Trochida	Tegulidae
Solitary	Nudibranchia	Mollusca	Gastropoda	Heterobranchia	Nudibranchia	n/a
Solitary	Unknown invertebrates	n/a	n/a	n/a	n/a	n/a
Encrusting	Demospongia	Porifera	Demospongia	n/a	n/a	n/a
Encrusting	*Distaplia* spp.	Chordata	Ascidiacea	n/a	Aplousobranchia	Holozoidea
Encrusting	Unknown ascidians	n/a	n/a	n/a	n/a	n/a
Encrusting	Bryozoans	Bryozoa	Gymnolaemata	n/a	Cheilostomatida	n/a
Encrusting	*Watersipora* spp.	Bryozoa	Gymnolaemata	n/a	Cheilostomatida	Watersiporidae
Encrusting	*Lichenopora* spp.	Bryozoa	Stenolaemata	n/a	Cyclostomatida	n/a
Aborescent	Pooled	n/a	n/a	n/a	n/a	n/a

Photographs were used to enumerate 23 consensus taxa (Table 1). Solitary organisms were counted, encrusting groups were quantified through percentage cover, and arborescent organisms were quantified through the summation of their length and widths. Solitary organisms were counted (organisms tile^−1^) by screening images in Adobe Photoshop (CS5.1) with magnifications to the limit of the pixel resolution. Colonial/encrusting organisms were quantified using Image J software (Abràmoff, Magalhães & Ram, 2004), in which organisms were outlined and their areas measured (mm^2^) using the width of the tile as a scale. Abundance of each colonial/encrusting taxon was summed across like organisms by tile, and expressed as percentage cover. Arborescent organisms also were quantified using Image J, using the greatest length and width (cm) that were summed across all arborescent organisms by tile to generate a single measure (cm) of linear abundance.

### Environmental conditions

Environmental conditions during tile immersions were evaluated through seawater temperature and the concentration of subsurface chlorophyll a. Temperature was chosen because of its broad effects on organism performance (Angilletta, 2009), and chlorophyll a because it provides a measure of the availability of particulate food (Paulay, Boring & Strathmann, 1985). Seawater temperature was accessed from NOAA buoy 46222 (0.01 °C resolution for Waverider Buoy) which is 26 km east of Santa Catalina Island and reports temperature from 0.5 m depth at 0.0006 Hz (Fig. S1, Supplemental Material). Records were averaged by day, and used to characterize the calendar year of each tile deployment, as well as the immersion time of the tiles. Using days as replicates, the thermal regimes during immersions were characterized using eight variables: (a) mean, (b) standard error, (c) maximum, (d) minimum, (e) the number of days warmer than the 90^th^ percentile value (*i.e*., extreme warm days), (f) the number of days cooler than the 10^th^ percentile value (*i.e*., extreme cool days), (g) day of onset of the period of extreme warm days (based on a 365 day year (366 days in a leap year)), and (h) day of onset of the period of extreme cool days (based on a 365 day year (366 days in a leap year)). The percentile vales were calculated across the study, and were used to statistically identify thermally extreme days because there is no information on biologically meaningful extreme values for the diverse fauna detected on the settlement tiles.

Chlorophyll a concentrations (mg m^−3^) were obtained through remote sensing using the Aqua MODIS sensor (4 km resolution) accessed through the ERDAPP interface (https://coastwatch.pfeg.noaa.gov) for 2011–2021. On this platform, 8 day composite data were obtained for a 185 km × 110 km grid positioned with its southwest corner located at 33.020208°N − 120.02083°W. Chlorophyll a data were summarized by month and used to characterize the calendar year of each tile deployment.

### Statistical analyses

Biological data were summarized using descriptive statistics evaluating the abundance of solitary and colonial/encrusting taxa (pooled among taxa) using tiles as replicates. Descriptive statistics were calculated for each taxon for both functional groups, and the trends for the five most abundant taxa for solitary and colonial/encrusting groups are reported. Densities were compared among times using permutational ANOVAs (*i.e*., PERMANOVA) (Anderson, 2001) because data were not normally distributed for some taxa. PERMANOVAs were conducted using 999 permutations with Type III sum of squares, and pairwise contrasts between consecutive years were completed with permutational t-tests with exact permutational *P*-values (Anderson, Gorley & Clarke, 2008).

Multivariate composition and abundance of settlers on the tiles was evaluated using 2-dimensional ordinations, first for all groups (*i.e*., solitary, colonial/encrusting, and arborescent combined), then separately for solitary and colonial/encrusting taxa. Data were standardized (*i.e*., z-transformed) for the pooled analyses because taxa were measured on different scales, and ordinations were prepared using non-metric multi dimensional scaling (MDS) (Clarke & Gorley, 2006) with resemblance matrices calculated using Bray-Curtis dissimilarities. Data were transformed prior to ordinations to adjust for the influence of extreme values, with the stringencies of the transformations adjusted (from for square root to log(X + 1)) for more varied data to reduce the stress of the ordinations.

Prior to testing for associations with environmental conditions, the measures of environmental condition were screened for collinearity, and variables excluded when associations were strong, unless a compelling biological reason suggested they should be retained. Tests of association were conducted in two ways, first using Pearson correlations by taxon, and second, using the BEST routine in Primer 6 to evaluate the extent to which biotic data (abundance and cover of taxa on the tiles) could be explained by environmental data. BEST analyses were conducted using the biotic data combined, and separately for the abundance of solitary taxa, and cover of encrusting/colonial taxa. Environmental data were selected for independence (*i.e*., without collinearity) and potential biological importance as described in results. Values were standardized because they were measured on different scales, and similarities were calculated using Euclidian distances. All combinations of variables were used, and significance was evaluated using 999 permutations with the rank correlation statistic, rho (Clarke & Gorley, 2006).

Univariate analyses were completed using Systat 13.0 software (Inpixon, Palo Alto, USA), and multivariate analyses were completed using Primer 6 (version 6.1.16) and PERMANOVA+ (version 1.0.6) (Primer-w; Quest Research Ltd, Auckland, New Zealand).

## Results

### Biological sampling

Ten tile deployments were completed, with the first beginning on 19 November 2010, and the last ending on 30 March 2021. Dates of deployments and retrievals varied, and immersion times ranged from 321 days (2012 and 2014) to 413 days (2011), with a mean of 366 ± 14 days (± SE, *n* = 10) (Fig. S1). There was no association between the abundance of fouling organisms in each of the three functional groups and immersion time (*P* ≥ 0.478, Fig. S2).

**Figure 1:**
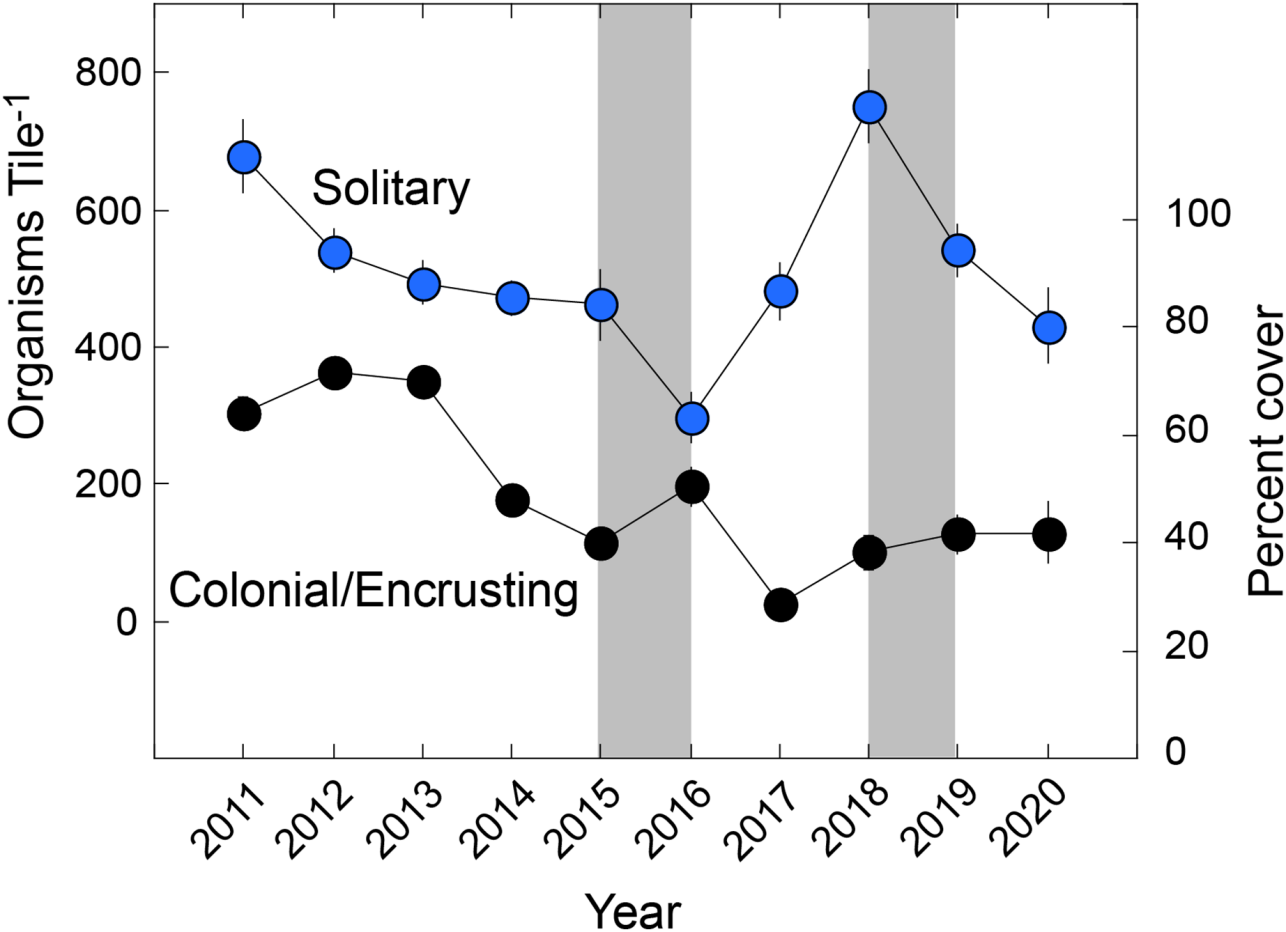
Organisms found on tiles by functional group. Summary of taxa on settlement tiles deployed from late 2010 to early 2021 (Year) and separated by solitary (scaled on left ordinate) and colonial/encrusting taxa (scaled on right ordinate). Symbols show mean ± SE (*n* = 15–19 y^−1^), with errors smaller than symbols where they are not visible. Grey bars show El Niños that generally each affect two sampling years.

The solitary fauna was dominated by *Spirorbis* spp. at a mean density of 239 ± 14 animals tile^−1^ (*n* = 154 tiles) (reaching 1,042 animals tile^−1^), and cirrepedian barnacles at 220 ± 11 animals tile^−1^ (reaching 659 animals tile^−1^). *Spirorbis* spp. accounted for 46.0% of solitary animals, cirrepedes 42.3%, solitary ascidians 4.7%, vermetids 2.8%, gastropods 1.3%, *Spirobranchus* spp. 1.3%, and all other groups combined, 1.7% (10 groups). The colonial/encrusting fauna covered a mean of 50 ± 2% of the tiles, and was composed of bryozoans (21.7 ± 1.3% cover), *Watersipora* spp. (14.5 ± 0.8%), Demospongia (9.0 ± 0.8%), *Lichenopora* spp. (2.0 ± 0.2%), social and compound ascidians (3.0 ± 0.3%), and *Distaplia* spp. (<0.1%). Aborescent taxa formed a tangled assemblage of bryozoans, fleshy macroalgae, hydrozoans, and articulated coralline algae, and their mean summed linear abundance was 69 ± 3 cm tile^−1^ (±SE). Analysis by functional group (Fig. 1) showed that solitary taxa changed in abundance (Pseudo-F = 16.535, df = 9,153, P_perm_ = 0.001), declining from a mean of 679 ± 53 animals Tile^−1^ in 2010 to 431 ± 54 animals Tile^−1^ in 2020, but with a minimum density of 298 ± 38 animals Tile^−1^ in 2016, and maximum of 751 ± 53 animals Tile^−1^ in 2016. Colonial/encrusting taxa changed in abundance (Pseudo-F = 22.756, df = 9,144, P_perm_ = 0.001), declining in cover from 64 ± 3% in 2010 to 42 ± 6% in 2020. Arborescent taxa changed in abundance (Pseudo-F = 30.511, df = 9,144, P_perm_ = 0.001), and declining from a mean summed dimension of 64 cm to in 2011 to 25 cm in 2020. The mean abundance of solitary taxa and encrusting taxa were not linearly related with time (F ≥ 0.108, df = 1,8, *P* ≥ 0.750), but the abundance of colonial/encrusting taxa declined linearly with time (F = 12.420, df = 1,8, *P* = 0.008).

**Figure 2:**
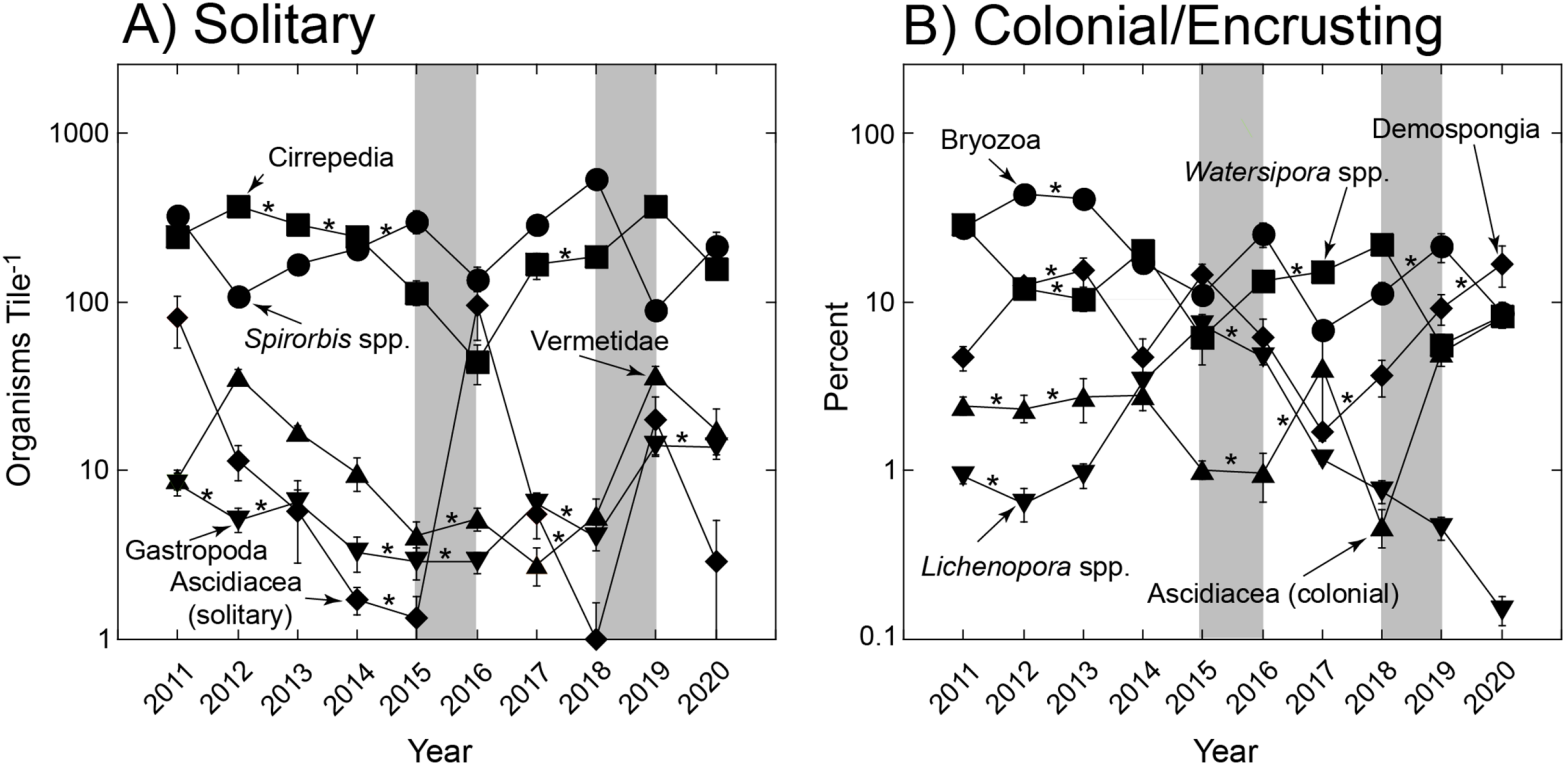
Most common organisms found on tiles. Summary of variation in abundance of solitary taxa (A) and colonial encrusting taxa (B) on tiles deployed for multiple immersion periods of ~1 yr from late 2010 to early 2021. Symbols show mean ± SE (*n* = 15–19 y^−1^), with errors smaller than symbols where they are not visible. Refer to Table 1 for a summary of taxa that were scored. Grey bars show El Niños that generally each affect two sampling years, and asterisks show sequential years in which abundances did not differ as detected through paired contrasts within a PERMANOVA framework (Table 1); abundances differ between unmarked pairs of consecutive years.

The trends in abundance of solitary and colonial/encrusting taxa (Fig. 1) were emergent features of diverse origins with respect to the five most common taxa in each group (Fig. 2). Among solitary taxa, the changes in abundance of *Spirorbis* spp. and barnacles between 2011 and 2020 were small, but both showed depressed abundances in 2016 that quickly were reversed, and similarly for *Spirorbis* spp. in 2019; the abundances of vermetid gastropods and other gastropods declined from 2012 to about 2016/17 before increasing to 2011; and the abundances of solitary ascidians generally declined, but this trend was punctuated by two transitory increases in 2016 and 2019 (Fig. 2A). The abundances of colonial/encrusting taxa were more dynamic, and all five of the common taxa showed large changes in abundance over time (Fig. 2B). *Watersipora* spp. and other bryozoans declined in cover, Demospongia and ascidians showed strong variation among years but were more abundant in 2020 than 2011, and *Lichenopora* spp. increased in cover from 2011 to 2015, before virtually disappearing by 2020. All common taxa (of both functional groups) significantly differed over time (Table 2).

**Table 2:** Environmental conditions during immersion periods of the settlement tiles. (A) Mean daily seawater temperature (±SE) during immersion years for each deployment of tiles (as shown in Fig. 1S) that varied in duration from 321 days (2012) to 419 days (2015). Red dots = hottest daily maximum temperature recorded during the immersion period, blue dots = coolest daily minimum temperature recorded during the immersion period; asterisks show years affected by El Niño events. (B) Mean monthly chlorophyll a concentration (±SE, *n* = 12 months) during calendar years sampled by each deployment of settlement tiles (see Fig. S1).

Functional group	Taxon	Statistical contrast
Solitary	*Spirorbis* spp.	Pseudo F = 14.418, df = 9,144, P_perm_ < 0.001
	Cirripedia	Pseudo F = 29.778, df = 9,144, P_perm_ < 0.001
	Ascidiacea	Pseudo F = 9.178, df = 9,144, P_perm_ < 0.001
	Vermetidae	Pseudo F = 13.333, df = 9,144, P_perm_ < 0.001
	Gastropoda	Pseudo F = 8.217, df = 9,144, P_perm_ < 0.001
Encrusting	Bryozoa	Pseudo F = 23.369, df = 9,144, P_perm_ < 0.001
	*Watersipora* spp.	Pseudo F = 13.358, df = 9,144, P_perm_ < 0.001
	Demospongia	Pseudo F = 12.389 df = 9,144, P_perm_ < 0.001
	Ascidiacea	Pseudo F = 11.519, df = 9,144, P_perm_ < 0.001
	*Lichenopora* spp.	Pseudo F = 28.627, df = 9,144, P_perm_ < 0.001

**Figure 3:**
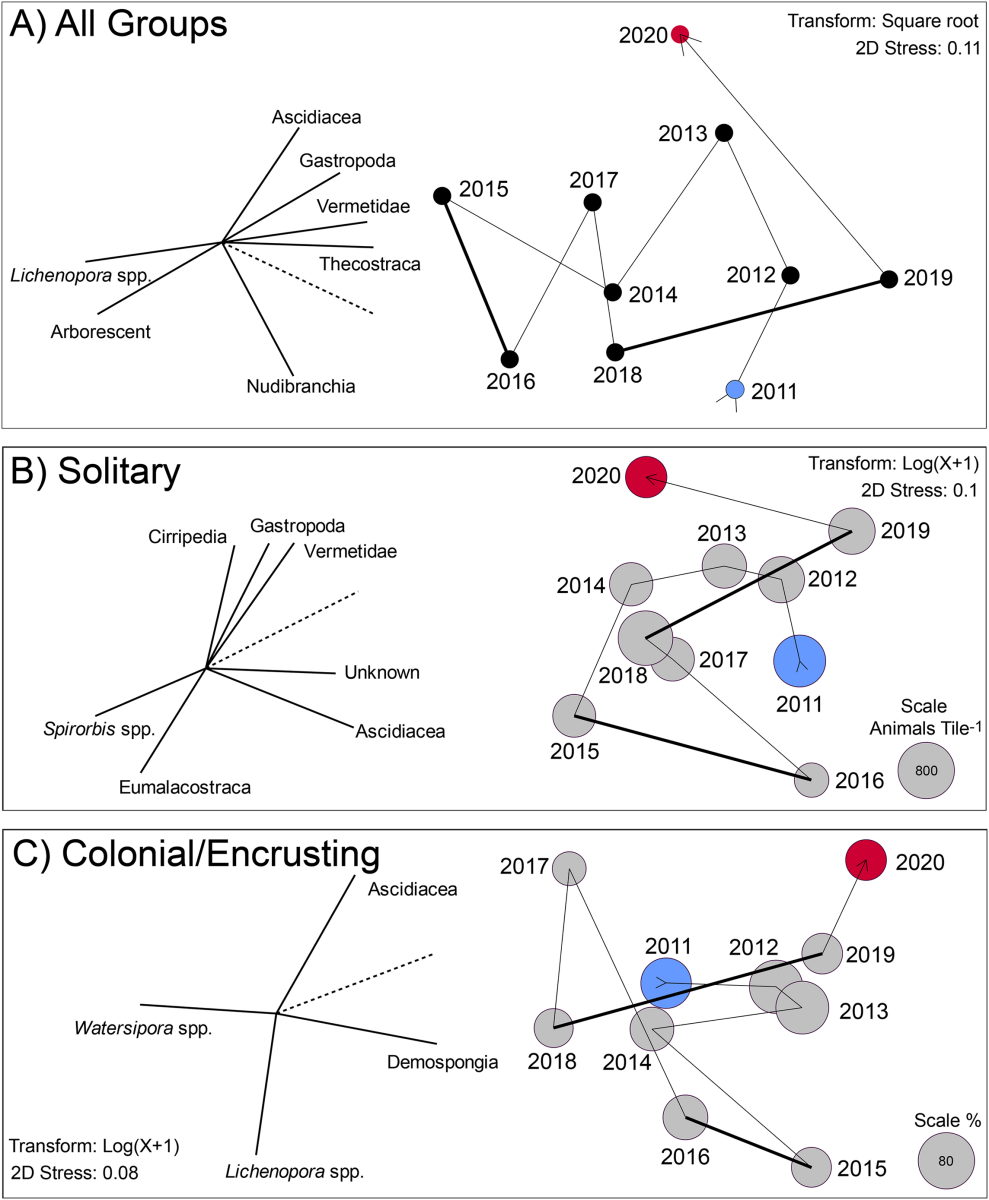
Two dimensional ordination describing change in communities developing on tiles immersed for approximately year-long periods from late 2010 to early 2021. (A) Analysis based on all groups scored on the tiles: solitary (16 taxa), encrusting (6 taxa), and arborescent (1 group) taxa. Values were standardized (z-score), square root transformed, and similarities calculated by the Bray Curtis method. Symbols labeled by immersion year (See Fig. S1), and vectors show role of the most influential taxa in separating years in two dimensions (screened to show strong relationships with r > 0.8, with dashed line scaling a value of 1.0). (B) Analysis based on solitary taxa, log(x + 1) transformed, with similarities calculated by Bray Curtis. Symbols are sized in area to represent overall density of this functional group, with scale in the bottom right of the plot. Vectors show role of the most influential taxa in separating years in two dimensions (screened to show strong relationships with r > 0.7, with dashed line scaling a value of 1.0). (C) Analysis based on colonial/encrusting taxa, log(x + 1) transformed, with similarities calculated by the Bray Curtis method. Symbols are sized in area to represent overall percentage cover of this functional group, with the scale in the bottom right of the plot. For all sampling symbols, blue is the first year, and red is the last year. Vectors show role of the most influential taxa in separating years in two dimensions (screened to show strong relationships with r > 0.7, with dashed line scaling a value of 1.0). Thick lines in all three MDS plots link periods of El Niño influence.

The multivariate changes in the invertebrates on the tiles are captured by 2D ordinations in which the stress values were ≤0.11, indicating the ordinations statistically had good repeatability (Fig. 3). All three ordinations show the extent to which the settlers departed from the initial condition by 2015 or 2016, and remained different in 2020, but for different reasons (based on the spatial location of 2020 *vs*. 2015 in the ordinations). The vectors on these plots reveal the extent to which the abundances of the component taxa were associated (Pearson correlations) with the values on the two MDS axes. Vectors approaching 1.0 indicate a strong association for taxa with a big effect in distinguishing among years in X and Y ordination space. For the pooled groups (*i.e*., solitary + colonial/encrusting + encrusting), the community changed from 2011 to 2015 through increases in *Lichenopora* spp. and arborescent taxa, and reductions in ascidians, gastropods, vermetids and barnacles (revealed by the length and direction of the vectors); these trends reversed from 2015 to 2020 (Fig. 3A). For solitary taxa, the community changed from 2011 to 2015 through increases in *Spirorbis* spp. and eumalacostracans, and reductions in gastropods, vermetids, and unknowns; these trends reversed from 2015 to 2019, although 2020 brought changes characterized by reductions in ascidians (Fig. 3B). For colonial/encrusting taxa, the community initially changed through increases in demosponges (2011–2013), then reductions in demosponges and increases in *Watersipora* spp. (2013–2018), and finally, increases in ascidians and demosponges with reductions in *Watersipora* spp. (2018–2020) (Fig. 3C).

### Environmental conditions

**Figure 4:**
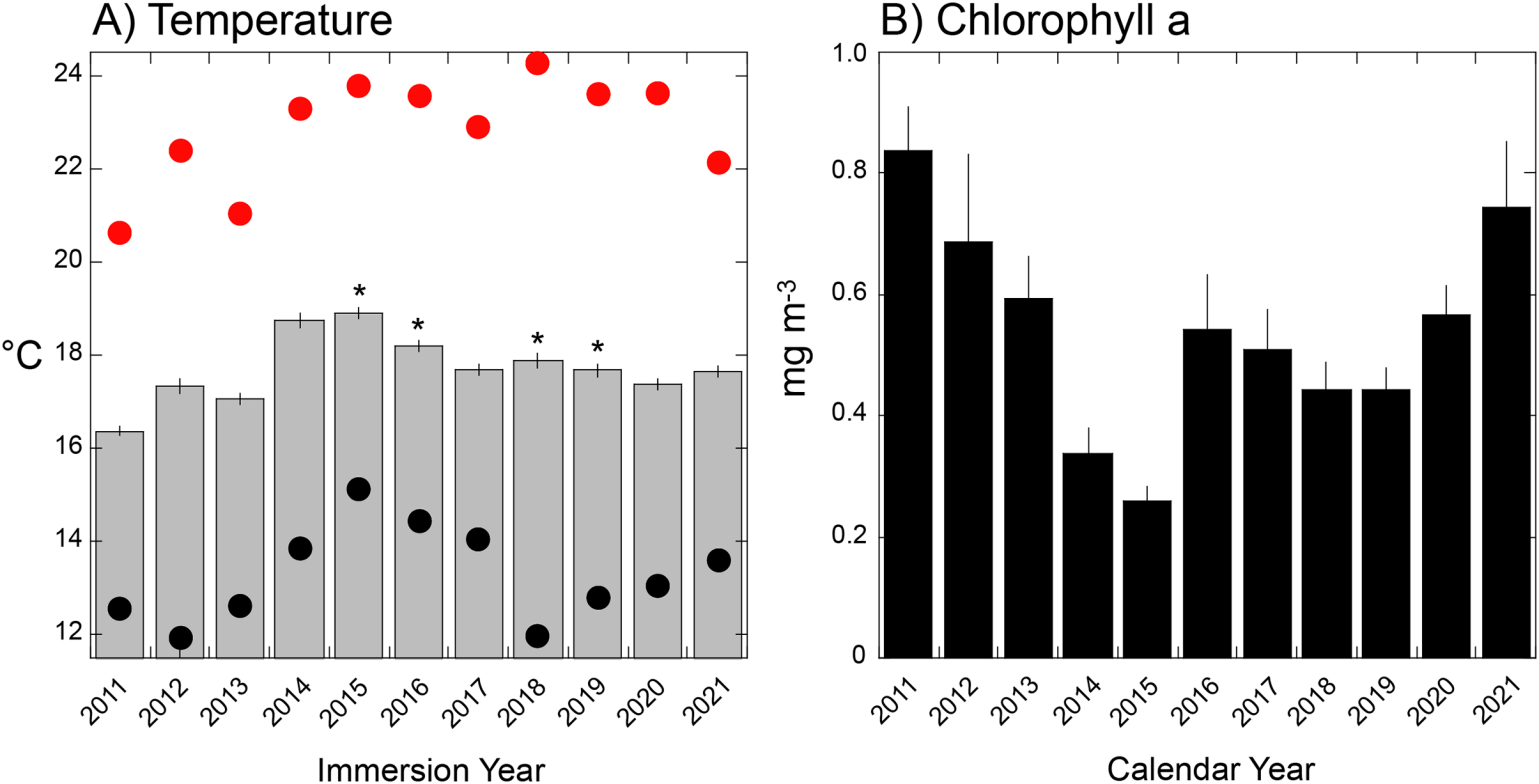
Environmental conditions during immersion periods of the settlement tiles. (A) Mean daily seawater temperature (±SE) during immersion years for each deployment of tiles (as shown in Fig. 1S) that varied in duration from 321 days (2012) to 419 days (2015). Red dots = hottest daily maximum temperature recorded during the immersion period, black dots = coolest daily minimum temperature recorded during the immersion period; asterisks show years affected by El Niño events. (B) Mean monthly chlorophyll a concentration (±SE, *n* = 12 months) during calendar years sampled by each deployment of settlement tiles (see Fig. S1).

Seawater temperature varied seasonally from a long-term winter mean low of 14.8 °C in early March, to a mean summer high of 21.0 °C in early September. Thermal variance (SE based on annual variation among days) was greatest in the summer (0.61 °C, early September) and lowest in late spring (0.15 °C, early June) (Fig. 4A). Mean temperature during tile immersion was lowest in 2011 (16.7 ± 0.1 °C), and highest in 2015 (19.3 ± 0.1 °C), with four of five warmest years corresponding to El Niños (and the fifth, 2014, to a warm water anomaly). Mean temperature differed among years (F = 2.26 × 10^17^, df = 10,4015, *P* < 0.001), but it did not change linearly with time (regression: F = 0.790, df = 1,8, *P* = 0.400). The distribution of thermally extreme days differed from the mean, with the hottest day in 2018 (24.3 °C, August 9^th^) and the coldest in 2012 (11.9 °C, March 19^th^) and 2018 (12.0 °C, April 17^th^). Analysis of daily seawater temperature throughout the study revealed the 10^th^ percentile at 14.59 °C, and 90^th^ percentile at 21.13 °C, and these extreme values identified between 0 days (in 2015) and 91 days (2011) in the lower tail, and between 0 days (2011) and 105 days (2015) in the upper tail, based on immersion years (Table S1, Fig. S1). When extreme cool periods occurred, they started between day 1 (*i.e*., 1 January as in 2011) and day 108 (*i.e*., 16 April as in 2018) in each calendar year, with extreme warm periods starting between day 190 (*i.e*., 7 July as in 2018) and day 239 (*i.e*., 25 August as in 2012 Table S1).

**Figure 5:**
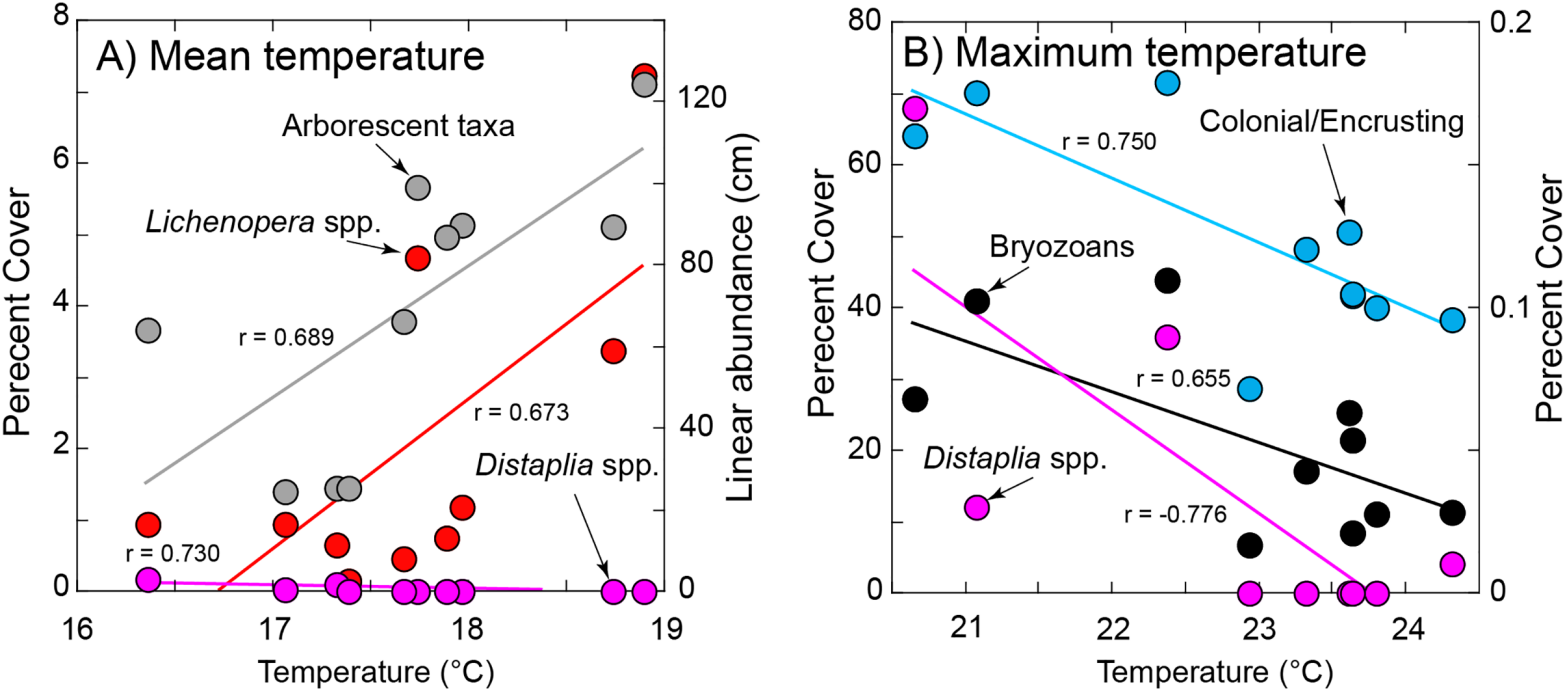
Significant relationships between organism abundance and seawater temperature during immersion. (A) Relationships of percentage cover of *Lichenopora* spp. and *Distaplia* spp. (left ordinate) and linear abundance of arborescent taxa (right ordinate) against mean daily temperature during immersion. (B) Relationships of percentage cover colonial/encrusting taxa and bryozoans (left ordinate) and *Distaplia* spp. (right ordinate) against maximum daily temperature during immersion. Lines in both panels show significant model I linear regressions (*P* ≤ 0.044) with correlation coefficients (r) next to each line. Symbols correspond to taxa as labeled.

Mean (±SE, *n* = 12) monthly chlorophyll a varied from 0.26 ± 0.02 mg m^−3^ in 2015 to 0.84 ± 0.07 mg m^−3^ in 2011 (Fig. 5B), and differed among years (F = 5.119, df = 10,120, *P* < 0.001). The four lowest concentrations occurred in the El Niños of 2015–2016, 2018–2019, and corresponded to years of high mean seawater temperatures (Fig. 5A).

Of the eight variables describing temperature (Table S1) four (minimum, days >90^th^ percentile, days <10^th^ percentile, day of onset of <10^th^ percentile) were correlated with mean seawater temperature (r ≥ 0.670, df = 5–8, *P* ≤ 0.034) and excluded from tests of association with organism abundance. The maximum and mean temperatures also were correlated (r = 0.725, df = 10, *P* = 0.018), but both were retained in the analysis because it reasonable to expect organisms might be responsive to both aspects of temperature. Chlorophyll a and temperature were correlated (r = −0.947, df = 8, *P* < 0.001), with high temperature corresponding to low concentrations of chlorophyll a; chlorophyll a was excluded from tests of association with organism abundance.

The abundance of *Lichenopora* spp., and arborescent taxa increased with mean temperature during immersion (r ≥ 0.673, df = 8, *P* ≤ 0.033), and the abundances of the remaining groups were unrelated to mean temperature (r < |0.588|, df = 8, *P* ≥ 0.118) (Fig. 5). The percentage cover of bryozoans, *Distaplia* and encrusting taxa (combined) were negatively associated with the maximum daily temperature during immersion (r ≤ −0.655, df = 8, *P* ≤ 0.044), but there were no other significant associations (r < |0.488|, df = 8, *P* > 0.153) (Fig. 5). The abundance of *Spirorbis* spp. worms was negatively associated with day of onset of extreme warm temperature (defined by the 90^th^ percentile cut-off of 21.13 °C (r = −0.709, df = 6, *P* = 0.049), showing that large numbers of these worms occurred on tiles immersed in years when seawater warmed early in the year. There were no other significant associations with the timing of onset of warm seawater (r = |0.633|, df = 6, *P* ≥ 0.092). The abundance of *Aplysia* spp. was positively associated with the SE of daily temperature (r = 0.680, df = 8, *P* = 0.030), but no other associations of abundance or percentage cover were significant (r < |0.442|, df = 8, *P* ≥ 0.201)). BEST analyses were conducted with same four measures of seawater temperature, which supported inclusion of only 8 years (as extreme warm days did not occur in 2011 or 2013). There were no associations of any combinations of environmental conditions and all taxa (ϱ = 0.116, P_perm_ = 0.64), solitary taxa (ϱ = 0.158, P_perm_ = 0.68), or encrusting taxa (ϱ = 0.077, P_perm_ = 0.85) on the tiles.

## Discussion

Analyses of fouling communities have been a staple of marine research for over a century (Visscher, 1928), with numerous studies describing the organisms, the rate and sequence at which they settle, and how these events vary over time (*e.g*., Sutherland, 1974; Menge et al., 2011 and references therein). Through the enumeration of organisms on surfaces immersed in seawater, advances have been made in understanding fundamental processes organizing benthic communities, including the roles of competition (Jackson, 1977), succession (Sutherland & Karlson, 1977), and disturbances (terHorst & Dudgeon, 2009); some of these discoveries have come from tiles deployed in the same ecosystem as studied herein (Keough & Downes, 1982; Broitman, Blanchette & Gaines, 2005; Bram, Page & Dugan, 2005; terHorst & Dudgeon, 2009). Against this backdrop, aspects of the present results are predictable: recruitment varied among years (cf. Vance, 1988; Bram, Page & Dugan, 2005), solitary taxa were dominated by spirorbid worms and barnacles, and encrusting taxa were dominated by bryozoans and sponges (cf. Scheer, 1945; Keough & Downes, 1982, Bram, Page & Dugan, 2005). However, sustaining tile deployments for a decade is unusual (cf. Keough & Downes, 1982; Bram, Page & Dugan, 2005; but see Menge et al., 2011 for an exception), and while even this duration had a limited capacity to support tests of associations between recruitment and environmental conditions, it revealed a decline in abundance of encrusting taxa, and a high degree of temporal variation that differed among taxa that together support two conclusions. First, by revealing a decline in abundance of a functional group of organisms that historically has dominated space on settlement tiles in shallow marine environments, our study suggests these communities may be poised for changes driven by alleviation of competition for space. Below we speculate as to the causes of this trend. Second, by revealing large variation in recruitment among years within a decade-long study, it is clear that caution must be placed in interpreting trends revealed by the common deployment of settlement tiles in studies lasting only a few years.

The taxa recorded on the present settlement tiles share strong similarities with those recorded on similar surfaces deployed in California seawater for decades. For example, through surveys completed from 1943–1945, fouling organisms were described in Newport Harbor, 60 km east of the present study site, thus revealing communities dominated either by algae, bryozoans, the solitary tunicates *Ciona* or *Styela*, or the mussel *Mytilus* spp. (Scheer, 1945). These communities were beneath docks, and the differences were attributed to succession mediated by immersion duration, and season of first immersion; *Mytilus* was considered the climax stage. Over 1979–80, Breitburg (1985) found that caged plates at 15 m depth off Naples Reef (24 km west of Santa Barbara) were colonized over 14 months by filamentous red algae (~8% cover), bryozoans (~20–30%), barnacles (~5%), tubiculous polychaetes (~5–20%), sedentary polychaetes (8–22%), and hydroids (~10%), with uncaged plates dominated by algal crusts, diatoms, algal films, and filamentous algae. Over 1999–2001, Bram, Page & Dugan (2005) deployed settlement tiles at 6–12 m depth for 2–24 months, 7 km west of Carpinteria, and their tiles were colonized mostly by a colonial tunicate, encrusting bryozoans, tubiculous amphipods, barnacles, and sponges (but not macroalgae). As described by Scheer (1945), taxa appeared in sequence, with colonial tunicates settling first, followed by bryozoans, then barnacles and sponges, and finally, with *Mytilus* appearing after 12 months. Bram, Page & Dugan (2005) suggested this was not classical succession (*sensu* Clements, 1936), rather it was a product of the biotic features of colonists modulated by settlement time and environmental conditions.

Within 500 m of the present study, Keough & Downes (1982) deployed settlement tiles at 10 m depth in 1981, and 3–4 weeks later found them colonized by the polychaete *Spirorbis* sp. and three species of bryozoans. Further away (750 m) at Bird Rock, Vance (1988) monitored fouling communities on rock walls at 12–17 m depth, and compared cleared and uncleared (control) quadrats for 4.5 years, starting in 1975. Cleared quadrats quickly reestablished a community comparable to controls, and at the end of the study, both were spatially dominated by ascidians (~10% cover), erect bryozoans (~5–10%), anthozoans (~5%), sponges (~10%), and rhodophytes (~10–20%). The encrusting bryozoan *Parasmittina* sp. initially colonized cleared quadrats at high coverage (38–50% cover), but did not persist, and *Mytilus* spp. (cf. Scheer, 1945; Bram, Page & Dugan, 2005) was not recorded.

The present tiles were submerged annually from 2010 to 2021, and they sampled propagules originating from the ~503 invertebrates and ~174 macroalgae recorded within the MPA in which the study occurred (Looby & Ginsburg, 2021), and probably also from more distant sources. Together, recruits varied among years, which included the strong El Niño of 2015–2016, the overlapping marine heat wave of 2014–2016, and the weaker El Niño of 2018 (Spiecker & Menge, 2022; Walker et al., 2020), when seawater temperature was elevated, and nutrients and chlorophyll a concentrations were low (Chavez et al., 2002, see also Fig. 4). During the study, the annual variation in recruitment appears idiosyncratic, with declines in abundance of some taxa occurring 1 year, followed by reversals the next, and visa versa (*e.g*., Fig. 2). The fauna on the tiles was numerically dominated by the same taxa each year (*e.g*., *Spirorbis* and barnacles (solitary), and bryozoans (encrusting)), the abundances of which substantially varied among years, although it appeared modest on a logarithmic scale (Fig. 2). For the other common solitary taxa, vermetids and other gastropods declined in abundance in the middle of the study, coincident with the 2015/16 El Niño, and solitary ascidians recruited in large numbers towards the end of both El Niños. For the other common colonial/encrusting taxa, *Lichenopora* spp. responded positively to the El Niño of 2015–2016, whereas colonial ascidians responded negatively. These two trends highlight the challenges of elucidating causation for these effects, for both *Lichenopora* spp. and colonial ascidians belong to the suspension feeding guild, yet they responded in contrasting ways to conditions that likely depressed food supply (*i.e*., phytoplankton as evaluated by the chlorophyll concentration of seawater). Together, these effects emerged in the 2D ordinations (Fig. 3), which revealed only modest directional changes in community structure, and differences associated with the El Niños (thick lines in Fig. 3) that were not appreciably greater than differences between environmentally unremarkable years.

When the temporal variation by taxon was collapsed by functional group (Fig. 1), trends emerged showing that colonial/encrusting organisms declined in abundance (by 35%) between 2011 and 2021, while solitary organisms maintained relatively stable abundances. Most colonial/encrusting organisms in fouling communities grow through asexual proliferation of a modular design, which makes them strong spatial competitors, that favors spatial dominance (Jackson, 1977). Once dominant, colonial/encrusting organisms can strongly influence community dynamics (Buss & Jackson, 1979), at least until the next disturbance. In the present study, colonial/encrusting organisms covered a long-term mean of 50% of the tiles, thereby depriving other taxa of settlement locations and strongly affecting community structure. Given more time (*i.e*., >12 months) the tiles might have become dominated by *Mytilus* spp. as described by Bram, Page & Dugan (2005) and Scheer (1945), and suggested by the capacity for this mussel to recruit on the nearby shore when predators are excluded (Robles, 1987). Unless (or until) this occurs, the occupancy of space by colonial/encrusting taxa has ecological importance in preventing the settlement of other organisms. Attenuation of this trend, as occurred from 2010 to 2021, therefore is likely to alter the availability of space on these tiles, thus attenuating the classic stiff competition for this resource (Jackson, 1977). With differential availability of space on benthic surfaces compared to the recent past, trajectories of community development are likely to change. Such effects are likely to become more important under regimes of rising seawater temperature that will intensify in coming decades (Cheng et al., 2020), and potentially will favor invasive species over natives species in fouling communities (Sorte, Williams & Zerebecki, 2010).

Despite the decadal duration of this study, it proved statistically challenging to test for associations between organism abundance and environmental conditions with only 10 replicates (*i.e*., years). Further, it was uninformative to consider multiple aspects of seawater temperature and chlorophyll a concentration as causal agents of change, because of strong collinearity among these dependent variables. Nevertheless, there were several statistically significant associations of organism abundance with aspects of seawater temperature that implicated temperature as a factor contributing to changes in the fouling communities detected on the tiles. Notably, the abundance of one colonial/encrusting taxon and arborescent taxa increased with temperature, whereas the abundance of bryozoans, *Distaplia* and encrusting taxa declined with higher maximum daily temperature during the immersion year. Further, spirorbid worms were more common in years when warming started early in the year, and the motile sea slug *Aplysia* spp. was more common in years with greater thermal variability among days. These trends do not demonstrate causation, and they do not support temperature as a major cause of the long-term trend in abundance of colonial/encrusting taxa (Fig. 1), because mean temperature did not increase linearly with time. Instead, seawater temperature was warmest in the middle of the study in association with El Niño effects and anomalous warming in 2013–2015 (Ohman, 2018). However, the pervasive effects of temperature on organism performance (Angilletta, 2009; Somero, 2002, 2012), highlights that this physical condition is likely to be a factor contribute to differences among years in the organisms settling on the present tiles. This possibility is consistent with the strong effects on temperature on the dispersal and viability of pelagic propagules (O’Connor et al., 2007), which support recruitment on the present settlement tiles, the structure of marine fouling communities in California waters (Sorte, Williams & Zerebecki, 2010), and the growth of spirorbid worms (Ni et al., 2018) that appeared responsive to one aspect of seawater temperature in the present study.

It was beyond the scope of the present study to test for causal effects of temperature in mediating variation in recruitment, or to discriminate the effects of temperature from chlorophyll a concentrations. Such analyses remain premature even after more than a decade of work on the current project, the modest number of replicate years provided for statistical analysis, and the experimental limitations of conducting research over lengthy periods. In this study, these effects are striking for the variation in immersion time of the tiles and the differences in dates of their deployment and retrieval (Fig. S1). While it remains desirable to alleviate these inconsistencies in future work, it is notable that the abundance of organisms on the tiles was not associated with their immersion duration. This outcome is likely to be a product of recruitment occurring as isolated events throughout each year (Broitman, Blanchette & Gaines, 2005; Menge et al., 2009, 2011), so that absolute immersion time matters less that being immersed for the relatively short period that the propagules are available in the seawater.

The study arose as a modest effort designed with the objective of being sustainable for decades, but achieving this objective was traded against a coarse taxonomic resolution and limitations of analytical capacity. Nonetheless, the temporal variability revealed by the data underscores the challenges of interpreting higher resolution studies that are conducted for a few years. A conclusion of the present study – that colonial/encrusting taxa have declined in percentage coverage – has interesting implications given the role of this functional group in fouling communities (Jackson, 1977). Since this functional group historically has dominated space on hard surfaces (Jackson, 1977), a reduction in this ability is likely to alleviate competition for this critical resource (*i.e*., space). An outcome of this trend would be to allow weaker spatial competitors to proliferate, and perhaps play a greater role in determining the functional attributes of fouling community (*e.g*., productivity and habitat provisioning). Since the present results also showed that colonial/encrusting taxa were less common in years characterized by a high maximum temperature, as might occur during a short marine heat wave, long-term increases in seawater temperature associated with climate change (Cheng et al., 2020) are likely to affect marine fouling communities through this potential mechanism. Indeed, using mostly colonial/encrusting taxa from the California subtidal, Sorte, Williams & Zerebecki (2010) experimentally demonstrated that consistent warming favors the growth of invasive *vs*. native taxa in fouling communities (cf. *Lichenopora* spp. and arborescent taxa in Fig. 5A). Together with the present analysis, these results suggest the structure of fouling communities in the future may depend on the response of recruiting taxa to thermal extremes (*i.e*., the maximum temperature) *vs*. the mean temperature. Analyses of these and other effects in marine fouling communities might benefit from triaging limited resources to maintain a larger number of decadal-scale, low-resolution analyses that are dovetailed with a smaller number of high-resolution studies conducted within a hypothesis driven framework.

## Supplemental Information

10.7717/peerj.14286/supp-1 Supplemental Information 1 The chlorophyll a concentration data.

10.7717/peerj.14286/supp-2 Supplemental Information 2 The biological data.

10.7717/peerj.14286/supp-3 Supplemental Information 3 The temperature data.

10.7717/peerj.14286/supp-4 Supplemental Information 4 Metadata for the three data files.

10.7717/peerj.14286/supp-5 Supplemental Information 5 Supplementary Information. Additional figures, data, and text in support of of the article.

## Additional Information and Declarations

### Data Availability

The following information was supplied regarding data availability: All the data used in this article are available in the Supplemental Files.
