# Supplementary material for: A decade of invertebrate recruitment at Santa Catalina Island, California
Source: PeerJ. 2022 Nov 8;10:e14286. doi: 10.7717/peerj.14286 (PMC9651044; doi:10.7717/peerj.14286)
Supplement: Supplemental Information 4 [file peerj-10-14286-s004.docx]

**A decade of invertebrate recruitment at Santa Catalina Island, California**

Peter J. Edmunds, Jessica Clayton

This document provides the metadata associated with the three .csv files containing all the data supporting this publication.

File: Chlorophyll Data

These data describe subsurface chlorophyll a concentration determined by remote sensing

• Chlorophyll a concentrations (mg m^-3^) were obtained through remote sensing using the Aqua MODIS sensor (4 km resolution) accessed through the ERDAPP interface (https://coastwatch.pfeg.noaa.gov). On this platform, 8 day composite data were obtained for a 185 x 110 km grid positioned with its southwest corner located at 33.020208°N -120.02083°W. Chlorophyll a data were summarized by month and used to characterize the calendar year of each tile deployment

Column A = sample year

Column B = sample month (1-12)

Column C = monthly average chlorophyll content (units mg m^-3^)

File: Biological Data

These data describe the organisms on the settlement tiles

Column A = sample year

Column B = tile number

Columns C-R = invertebrate abundance (listed in Table 1) as animals per tile

Columns S-X = cover of encrusting taxa (%, listed in Table 1) by tile

Column Y - summed linear dimensions (cm) of arborescent (fleshy) taxa

File: Temperature Data

These data obtained from NOAA Bouy 46222

• Seawater temperature was accessed from NOAA buoy 46222 which is 26 km east of Santa Catalina Island and reports temperature from 0.5 m depth at 0.0006 Hz. Records were averaged by day, and used to characterize the calendar year of each tile deployment, as well as the immersion time of the tiles.

Column A = sample month

Column B = month by number (1-12)

Column C = day by year (1-366)

Column D = day by month

Columns E-F = data by year 2010-2002 (°C)

nd = no data
