# Supplementary material for: A decade of invertebrate recruitment at Santa Catalina Island, California
Source: PeerJ. 2022 Nov 8;10:e14286. doi: 10.7717/peerj.14286 (PMC9651044; doi:10.7717/peerj.14286)
Supplement: Supplemental Information 5 — Additional figures, data, and text in support of of the article. [file peerj-10-14286-s005.docx]

SUPPLEMENTAL MATERIAL

**A decade of invertebrate recruitment at Santa Catalina Island, California**

Peter J. Edmunds, Jessica Clayton

*Deployment configurations*

Logistical constraints in the field resulted in the tiles being suspended at a fixed depth off a floating dock for three immersion years (2016-2018), beginning on 16 January 2016 and ending on 20 February 2019, and off a fixed (non-floating dock) for seven immersion years. When suspended off the fixed dock, the depth of the tiles slightly varied with tidal variation in seawater depth. Preliminary analyses of the fouling communities (averaged among tiles within each year) between these two periods revealed no difference between configurations for solitary taxa (t = 0.075, df = 8, P = 0.942), encrusting taxa (t = 1.595, df = 8, P = 0.149), or arborescent taxa (t = 1.393, df = 8, P = 0.201).

*2) Immersion times and temperature*

Logistical constraints prevented identical immersion times for the tiles, either in terms of absolute duration underwater (ranging from 321-419 days), and the start and end times of the immersions (Fig. S1). Tests of association between mean abundances (by sampling) for the three functional groups of fouling organisms and immersion time did not reveal significant effects (r ≤ 0.244, df = 8, P ≥ 0.478) (Fig. S2). Seawater temperature (averaged over 13 years) increased throughout the majority of each Immersion period (Fig. S1).


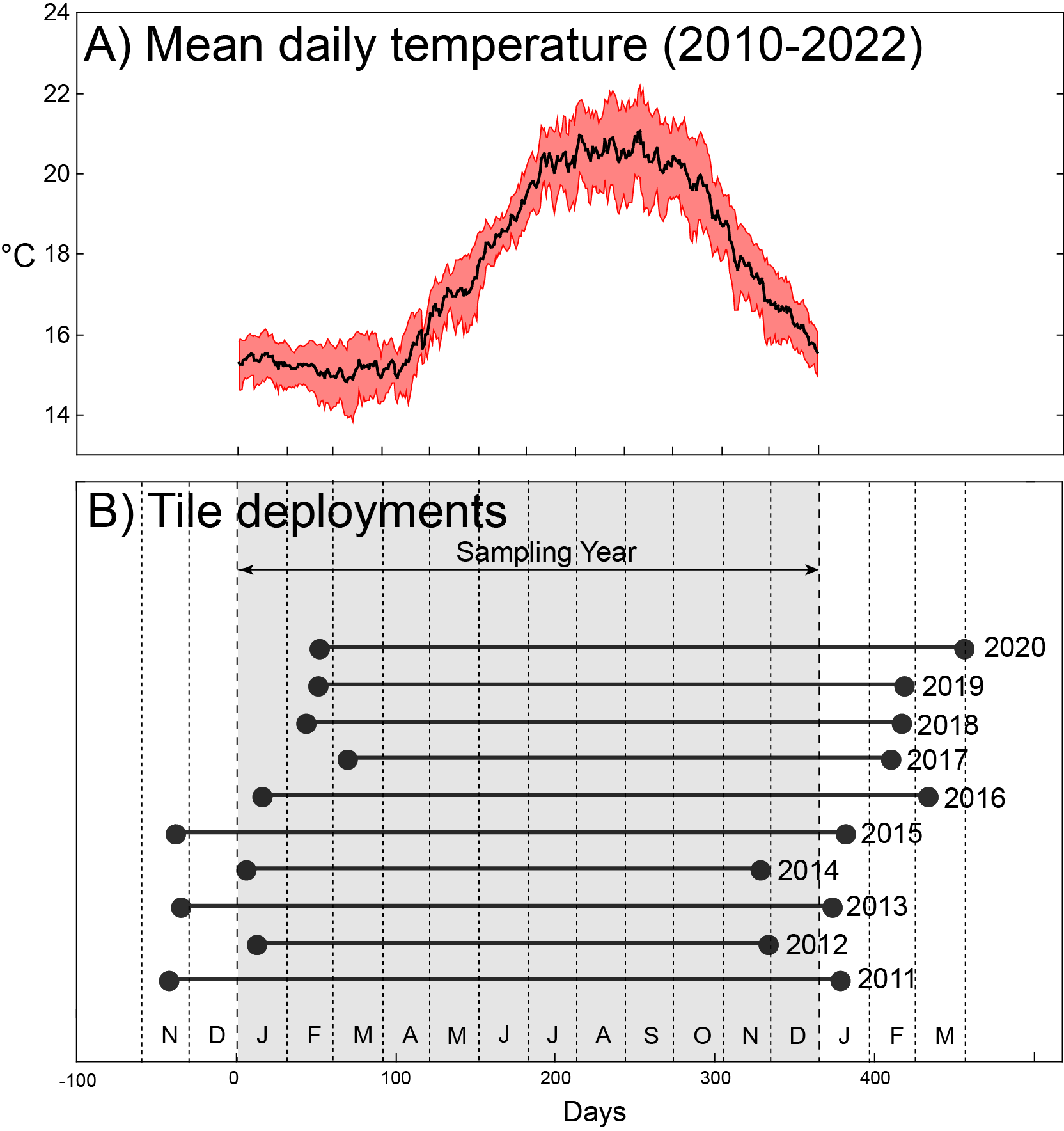


Fig. S1. **Seawater temperature (A) and sampling scheme (B).** Temperature shows mean daily records for NOAA buoy 46222 with red belt showing 95% confidence interval. Sampling scheme shows relationship of immersion year to calendar year. For some years, tiles were immersed prior to the start of the immersion year (negative days) and sometimes were retrieved early the following year.


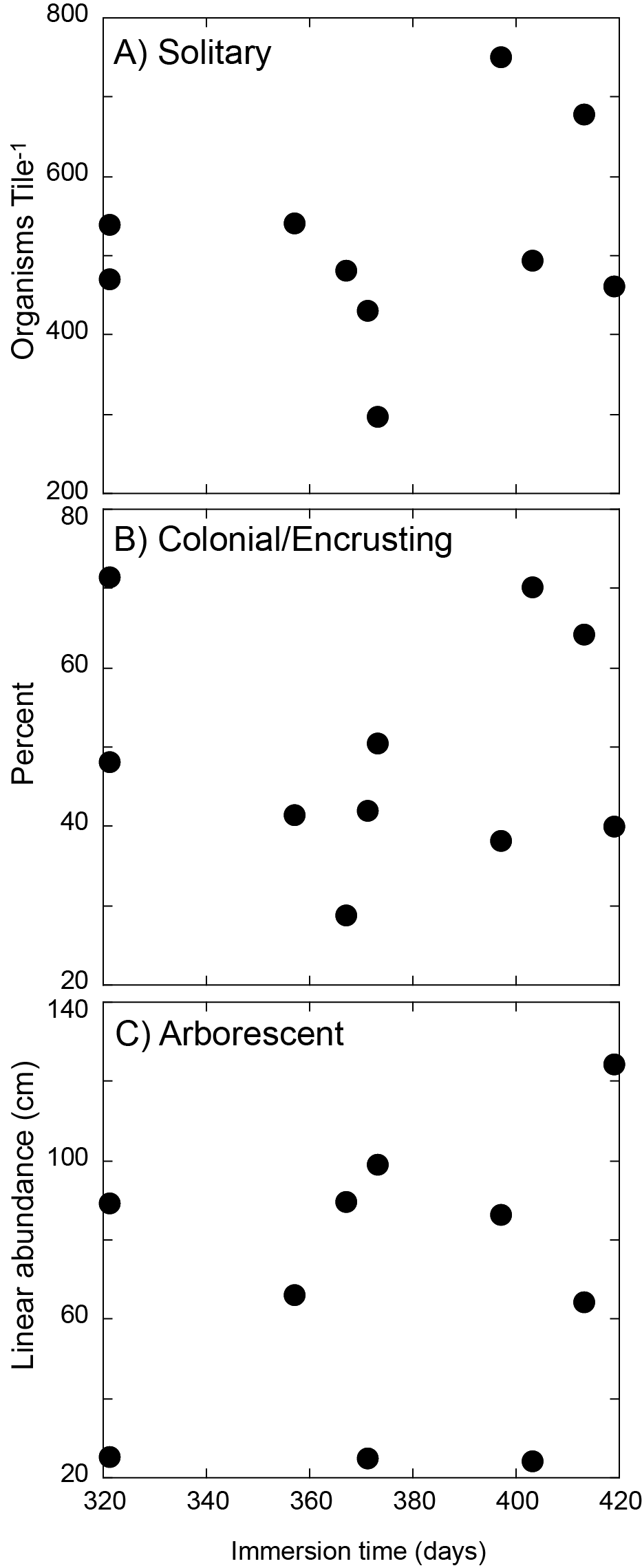


Fig. S2. Scatterplots showing relationships between the abundance of fouling organisms in three funtional groups (pooled among taxa) as a function of time immersion time (Fig. S1).

**Table S1. Summary of descriptive statistics for daily seawater temperature from 2010 to 2021.** Values for mean, standard error (SE), maximum (Max) and minimum (Min) are reported for immersion years (as in Fig. S1) with a variable number of days (No. days (N)) determined by the timing of tile deployment and recovery. The 10^th^ and 90^th^ percentile were defined across the whole study (14.59°C and 21.13°C, respectively) and the number of days in these tails as well as the onset of the extreme periods are reported for the main calendar year of immersion (Fig. S1). n/a = not applicable as extreme days (as defined here) did not occur in these years, or were rare and scattered throughout the year.

| Immersion Year | No. days (N) | Mean (°C) | SE (°C) | Max (°C) | Min (°C) | Days < 10^th^ percentile | Days > 90^th^ percentile | Day of onset of < 10^th^ percentile | Day of onset of > 90^th^ percentile |
| --- | --- | --- | --- | --- | --- | --- | --- | --- | --- |
| 2011 | 413 | 16.36 | 0.10 | 20.65 | 12.59 | 91 | 0 | 1 | n/a |
| 2012 | 321 | 17.33 | 0.15 | 22.37 | 11.92 | 71 | 28 | 45 | 239 |
| 2013 | 403 | 17.06 | 0.11 | 21.07 | 12.65 | 88 | 0 | 6 | n/a |
| 2014 | 321 | 18.74 | 0.15 | 23.31 | 13.88 | 9 | 71 | 93 | 238 |
| 2015 | 419 | 18.89 | 0.12 | 23.80 | 15.16 | 0 | 105 | n/a | 201 |
| 2016 | 416 | 17.73 | 0.11 | 23.61 | 14.08 | 18 | 49 | n/a | 205 |
| 2017 | 343 | 17.97 | 0.12 | 22.93 | 14.33 | 5 | 36 | 24 | 198 |
| 2018 | 373 | 17.89 | 0.15 | 24.31 | 12.01 | 35 | 60 | 55 | 190 |
| 2019 | 367 | 17.67 | 0.14 | 23.64 | 12.82 | 45 | 55 | 48 | 226 |
| 2020 | 398 | 17.38 | 0.12 | 23.64 | 13.08 | 3 | 21 | n/a | 230 |
| 2011 | 413 | 16.36 | 0.10 | 20.65 | 12.59 | 91 | 0 | 1 | n/a |
